# Antibacterial Efficacy of Different Concentrations of Sodium Hypochlorite Gel and Solution on *Enterococcus faecalis* Biofilm

**DOI:** 10.22037/iej.2016.11

**Published:** 2016

**Authors:** Vahid Zand, Mehrdad Lotfi, Mohammad Hosein Soroush, Amir Ardalan Abdollahi, Mehdi Sadeghi, Ali Mojadadi

**Affiliations:** a *Dental and Periodontal Research Center, Dental School, Tabriz University of Medical Sciences, Tabriz, Iran; *; b *Microbiology Department, Faculty of Medicine, Tabriz University of Medical Sciences, Tabriz, Iran; *; c *Dental and Periodontal Research Center, Department of Endodontics, Student Research Committee, Dental School, Tabriz University of Medical Sciences, Tabriz, Iran; *; d * Private Practice, Tabriz, Iran*

**Keywords:** Antibacterial, Biofilm, *Enterococcus Faecalis*, Sodium Hypochlorite

## Abstract

**Introduction:**

This *in vitro* study compared the antibacterial efficacy of 2.5% sodium hypochlorite gel and 2.5% and 5.25% sodium hypochlorite solutions on *Enterococcus faecalis* (*E. faecalis*) biofilm.

**Methods and Materials:**

The root canals of 60 extracted human single-rooted teeth were contaminated with *E. faecalis* and incubated for 6 weeks. The samples were randomly assigned to three experimental groups and one control group (*n*=15). The study protocol in the experimental groups consisted of injection of 5 mL of each irrigant into the root canals. Samples were collected from the root canal walls and 1:10 serial dilutions were prepared and added to Muller Hinton Agar (MHA) plates and incubated at 37^°^C for 48 h. A classic colony counting technique was used for determining vital *E. faecalis* bacterial counts in MHA plates. The Kruskal-Wallis test was used for statistical analysis of the data. The level of significance was set at 0.05.

**Results:**

The antibacterial effect of the irrigants in all three experimental groups was significantly greater than the control group (*P*<0.05), with no significant difference between 2.5% and 5.25% NaOCl solutions (*P*>0.05). The effect of 2.5% and 5.25% NaOCl solutions were significantly superior to 2.5% NaOCl gel (*P*<0.05).

**Conclusion:**

Under the limitations of this study, 2.5% NaOCl gel was effective in reducing *E. faecalis* counts; however this effect was less than that of NaOCl solutions.

## Introduction

It has been demonstrated that bacteria and their products have a vital role in initiation and progression of pulp and periapical diseases [1]. For this reason the principal objective of root canal treatment is to eliminate bacteria and their products from the root canal system [1-3]. Proper shaping and cleaning of root canal is a major prerequisite for successful root canal therapy [4]. However, anatomy of the root canal system, accessory root canals, apical ramifications and penetration of microorganisms deep into the dentinal tubules make it difficult and in some cases impossible to completely eliminate microorganisms from the root canal system only with instrumentation; in this context, irrigation with antimicrobial solutions is necessary [3, 5]. The bacteria existing in the biofilm show high degrees of resistance to antimicrobial agents because of the structure and physiology of the biofilm [6, 7]. Therefore, clinical studies have demonstrated that the bacterial biofilm may remain even after thorough mechanical and chemical preparation of the root canal system [3, 7]. 

*Enterococcus faecalis* (*E. faecalis*) is one of the most resistant microorganisms in the root canal system [1, 8] and is the most prevalent bacterial strain isolated from teeth with endodontic treatment failure [9]. In teeth with periapical periodontitis, *E. faecalis* has been found in 71% of the cases and its thorough elimination from the root canal is very difficult, if not impossible [3]. *E. faecalis* is able to produce biofilms in different conditions such as aerobic, anaerobic, rich or deficient nutrition [8]. 

Canal irrigation can disinfect regions that are not accessible to mechanical instrumentation. Sodium hypochlorite (NaOCl) is the most commonly used root canal irrigant [3, 5, 10]. However, there is controversy over its best concentration [11, 12]. NaOCl is used in concentrations from 0.5 to 5.25%, with its antimicrobial activity increasing proportionally; so does its toxicity [13]. NaOCl is effective against *E. faecalis* in all concentrations [3]. In addition, it can be used as a lubricant during root canal instrumentation [13]. The main disadvantage of NaOCl is its cytotoxic effects when it is extruded from the root canal into the periapical tissues because it can induce allergic reactions [11].

It is expected that using NaOCl gel can reduce the apical extrusion of debris and decrease its side effects. In addition, if the gel and solution forms are equality effective, the benefits of gel form in root canal treatment cannot be overlooked. Therefore, studies are necessary on the proper concentration of NaOCl and comparison between gel and solution forms. The aim of this *in vitro* study was to compare the antibacterial efficacy of 2.5% NaOCl gel and 2.5% and 5.25% NaOCl solutions on *E. faecalis* biofilms.

## Materials and Methods

The study was approved by the Research and Ethics Committee of Tabriz University of Medical Sciences. Sixty-five extracted human single-rooted teeth that were extracted because of periodontal disease were selected for this *in vitro* study. All the teeth had mature single straight roots, with no root caries, previous endodontic treatments and anomalies. In addition, the teeth exhibited no internal or external root resorption, calcifications and cracks or fractures. Subsequent to extraction, the teeth were stored in 3% chloramine-T solution at 4^°^C. The root surfaces were cleaned with ultrasonic tips to remove any residual periodontal soft tissues. The crowns were then dissected at the level of CEJ to achieve the root lengths of 12 mm. The working length was determined with a #15 K-Flexofile (Dentsply Maillefer, Ballaigues, Switzerland), 1 mm away from the apical foramen. Instrumentation was carried out with K-files up to #35, followed by preparation of the coronal two-thirds of the root canals with #4, 3, 2 and 1 Gates-Glidden drills (Dentsply, Maillefer, Ballaigues, Switzerland) using the crown-down technique. Each root canal was irrigated with 1 mL of normal saline during the instrumentation procedures. The smear layer was eliminated with 1 mL of 5.25% NaOCl (Taj Corp, Tehran, IRI) for 3 min, followed by irrigation with 1 mL of 17% ethylenediaminetetraacetic acid (EDTA, Pulpdent Corp, Watertown, MA, USA) for 3 min. The final irrigation was carried out with phosphate-buffered saline (PBS) solution. The teeth were sterilized in an autoclave at 121^°^C temperature and 15 psi pressure for 20 min.

***Microbiology procedures***

**Figure 1:**
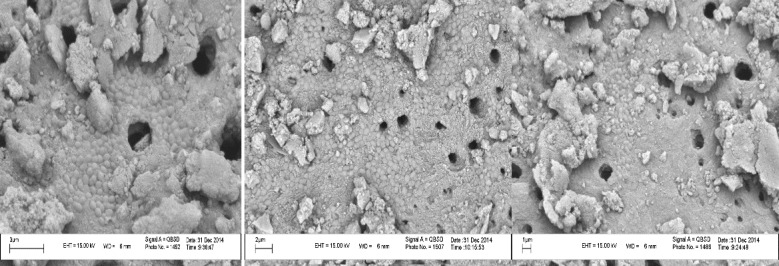
SEM micrographs of transverse sections of teeth to confirm the existence of *E. faecalis*

Each tooth was placed in a sterile micro-tube containing 2 mL of standard suspension of *E. faecalis* (ATCC 29212, Reference Laboratories of Iran Research Center, Tehran, Iran) [14]. This suspension was prepared in the Bacteriology Laboratory of Tabriz, School of Medicine. Bacterial count consisted of 1.5×10^8^ CFU/mL. The microorganisms were incubated for 6 weeks (to confirm the formation of mature biofilms) in brain-heart infusion broth (BHI, Merck, Darmstadt, Germany) at 37^°^C under aerobic conditions. During this period, BHI broth was changed every other day to maintain normal growth of *E. faecalis*. After that, 5 samples were selected randomly and sectioned with a diamond disk parallel to the long axis of the tooth up to the vicinity of the root canal. The teeth were bisected with a chisel in order to prevent accumulation of dentin chips. Then transverse sections of the teeth were provided and evaluated under a scanning electron microscope (SEM) (Figure 1). 

The remaining 60 samples were randomly divided into three experimental groups and one control group (*n*=15). Group 1, control; group 2, 2.5% NaOCl gel (consisted of 2.5% NaOCl, ethylene glycol and tri ethanolamine prepared by a pharmaceutics colleague); group 3, 2.5% NaOCl solution and group 4, 5.25% NaOCl solution. In the control group, no procedures were carried out until the sampling time and the bacteria were preserved in the incubator at 37^°^C. In groups 2, 3 and 4, a total of 5 mL of gel and solution test irrigants were injected into the canal and then the samples were incubated for 30 min [15]. Subsequently, the canals were irrigated with 0.6% sodium thiosulfate to neutralize the activity of NaOCl. All the teeth were frozen at -25^°^C in order to prevent *E. faecalis* from being killed due to the heat generated during the sampling phase. The efficacy of disinfection was evaluated by collecting dentin shavings due to drilling the walls of canals using #5 and 6 Gates-Glidden drills. The drills were inserted into the canals until they reached 1 mm short of the working length and 10 µg of dentin shavings were collected from each root canal. The samples were transferred into sterile tubes containing 2 mL of normal saline and vortexed for 20 sec. Serial 1:10 dilutions were provided. Then 100 µL of each solution was added to three Muller Hinton Agar plates and incubated at 37^°^C for 48 h. All the procedures were carried out in a laminar flow chamber with sterile instruments in order to observe the aseptic conditions. A classic colony counting technique was used for counting the vital *E. faecalis* bacteria in Muller Hinton Agar plates. The bacterial growth in agar plates related to the concentrations of 10^-5^, 10^-6^ and 10^-7 ^was not considered, because at concentrations higher than 10^-5 ^colony counting was not possible because of lack of bacterial overgrowth. Therefore there was no need to consider dilutions in groups 2, 3 and 4 and colony counting was possible at the first concentration, but in the control group colony counting was possible at 10^-3^ concentration.

Statistical analysis was performed using SPSS (Statistical Package for Social Science, SPSS, version 20.0, SPSS, Chicago, IL, USA). The Kolmogorov-Smirnov test showed that the data was non-parametric. Therefore, the Kruskal-Wallis test was used to compare the bacterial colony counts and the Mann-Whitney test was used for pairwise comparisons and therefore the amount of type *I* error (*α*=0.05) was set according to Bonferroni test which was obtained 0.008.

## Results

Table 1 presents the colony counts (CFU) in four groups (three experimental and one control groups) for different antimicrobial treatments. Quantitative data was reported as means, medians, 95% confidence interval, maximums and minimums. Since the colony counts (CFU) in groups 3 and 4 were zero (0), these groups are not presented in Table 1.

The results of Kolmogorov-Smirnov test showed the non-normal distribution of data in the evaluated groups. The results of Kruskal-Wallis test showed significant differences between the groups (*P*<0.05). The pairwise comparisons showed significant differences between 2.5% NaOCl gel and 2.5% NaOCl solution and 2.5% NaOCl gel and 5.25% NaOCl solution. Therefore, the difference between 2.5% NaOCl gel and 2.5% and 5.25% NaOCl solutions was significant but there was no significant difference between both solutions.

## Discussion

This study compared the *in vitro* antibacterial activity of 2.5% NaOCl gel, 5.25% and 2.5% NaOCl solutions against *E. faecalis.* *E. faecalis* was selected in the present study because it is one of the most resistant intracanal bacteria and the most common microorganisms isolated from teeth with persistent apical periodontitis [16, 17]. *E. faecalis* has a high rate of survival rate and high resistance to intracanal medicaments in biofilm and planktonic states [18]. Several studies have demonstrated that *E. faecalis* is highly capable of biofilm formation on human dentin after 72 h [19, 20]. In this study, the time period considered for confirmation of biofilm formation was 6 weeks because the calcified biofilm is seen in the six weeks [14] and according to Zand *et al.* [21], the bacteria in the old biofilms are more resistant to NaOCl than bacteria in young biofilms. 

An ideal intracanal irrigating solution should exhibit maximal antibacterial and tissue dissolving characteristics and the least toxic effects [22]. Sodium hypochlorite has been used as an endodontic irrigant for more than 70 years [23]. NaOCl is a potent dissolving agent for vital and necrotic tissues [24]. The potent antibacterial effect of NaOCl against *E. faecalis* depends on concentration and time of exposure. Although NaOCl is the most commonly used root canal irrigant because of its unique features, such as dissolving of organic tissue, killing of microorganisms and acting as a lubricant [25, 26], its toxic effects on vital tissues and induction of inflammatory reactions in case of over extrusion cannot be overlooked [27]. Since the NaOCl gel has lower risks of extrusion through the apex, in this study we compared the gel form of NaOCl with its solutions. Canal instrumentation and coronal flaring were carried out up to #35 K-files and #4 Gates-Glidden drills, which resulted in better penetration of irrigating solutions and gel into the root canals. Therefore, the lack of penetration of the solutions into the apical thirds of the canals would not be perceived as absence of antimicrobial activity.

The period of exposure to irrigating solutions and gel was selected according to the protocol suggested by Gomes *et al.* [11] and Neelaktan *et al.* [15]. The samples from the canal walls were collected using #5 and 6 Gates-Glidden drills. Evaluation of the dentin shavings formed by these drills allowed sampling from dentinal tubules and made it possible to study the penetration of irrigation solutions and gel into dentinal tubules.

NaOCl exhibits antibacterial effects against *E. faecalis* at different concentrations and exposure times; the 5.25% concentration is the most commonly used solution of NaOCl [24]. The reason for choosing 2.5% NaOCl in this study was that it has been shown to denature bacterial toxins and dissolve organic tissues [28-31]. Vaziri *et al.* [32] concluded that 2.5% NaOCl had a significant effect on the viability of *E. faecalis* and the antimicrobial efficacy by direct contact occurred after 2 min.

The results of the present study demonstrated no significant difference between the antimicrobial effects of NaOCl irrigation solutions at 2.5% and 5.25% concentrations on *E. faecalis*. These results concur with those reported by Siqueira *et al.* [33] and Zand *et al.* [31]. Contrary to our study, Berber *et al.* [34] showed that 5.25% concentration was the most effective solution followed by 2.5% concentration. In addition, a study by Sjögren *et al.* [35] showed that approximately 40% of the canal surfaces remain contaminated after irrigation with 2.5% NaOCl and this concentration is not appropriate for killing *E. faecalis*. The contradictory results of studies in relation to the antibacterial efficacy of different concentrations of NaOCl tested in this study might be attributed to differences in methodology, microbial characteristics in the biofilm, exposure time and concentration of the tested substance [36, 37]. 

This study showed that although 2.5% NaOCl gel was effective in killing *E. faecalis*, it exhibited significantly lower antimicrobial efficacy in comparison to 2.5% and 5.25% NaOCl irrigation solutions. Several studies have shown the potential of 2% chlorhexidine gel in eradicating *E. faecalis *[38]; also in addition, its advantages such as low toxicity to periapical tissues [39] and viscosity that keeps the active agent in contact with the root canal walls and dentinal tubules [40] have been reported. However, the effect of chlorhexidine in gel and solution forms on microbial biofilms is significantly less than that of NaOCl [41]. The lower antimicrobial efficacy of NaOCl gel in comparison to NaOCl solution could be probably attributed to the viscosity of gel and its inability to penetrate into the depth of dentinal tubules. 

**Table 1:** The CFUs in the 4 study groups

**Groups**	**Mean (SD)**	**Median**	**Min**	**Max**
**Control**	23 (100)	103000	11000	271000
**NaOCl gel**	21 (91.3)	1	0	17

This study was the first to evaluate the antibacterial efficacy of NaOCl in its gel form. Further studies should be carried out with different concentrations of NaOCl gel and with other bacteria. To determine the most effective root canal irrigator, the efficacy of the irrigating solutions and gels should be further determined with various bacterial species in root canals and with different methods. Further studies are needed to confirm the effect of findings of this study in clinical settings.

## Conclusion

Sodium hypochlorite irrigation solution at 2.5% and 5.25% concentrations resulted in the elimination of all the bacteria in 10 min while sodium hypochlorite gel did not exhibit the same effect with identical exposure time on *E. faecalis* biofilm.
